# The Influence of Graphene Content on the Antibacterial Properties of Polycaprolactone

**DOI:** 10.3390/ijms231810899

**Published:** 2022-09-17

**Authors:** Maciej B. Hajduga, Rafał Bobinski, Mieczysław Dutka, Jan Bujok, Michał Cwiertnia, Celina Pajak, Anna Kurowska, Izabella Rajzer

**Affiliations:** 1 Faculty of Health Sciences, ATH University of Bielsko-Biala, 43-309 Bielsko-Biała, Poland; 2 Faculty of Mechanical Engineering and Computer Science, ATH University of Bielsko-Biala, 43-309 Bielsko-Biała, Poland

**Keywords:** polycaprolactone, graphene, antibacterial, biomaterials

## Abstract

This work contains an analysis of the impact of modifying a bioresorbable polymer—polycaprolactone (PCL)—with various additives on its antibacterial properties. To this end, samples of PCL filament containing various content levels of graphene (GNP), 0.5%, 5%, 10%, were obtained using injection molding. Polymer samples without additives were used for comparison. The next step was to assess the antimicrobial impact of the preparations under study against the following microorganisms: *Staphylococcus aureus* ATCC 25293, *Escherichia coli* ATCC 25922, *Candida albicans* ATCC 10231. Effective bactericidal activity of PCL with small amount of GNP, especially against *C. albicans* and *S. aureus* was confirmed. A decrease in this property or even multiplication of microorganisms was observed in direct proportion to the graphene content in the samples.

## 1. Introduction

Polymers are a very important group of materials used in modern medicine. Polymers are used as scaffolding materials for artificial skin and tissue engineering. Polymers are also used in carrier formulations for transdermal and transdermal drug delivery. Polycaprolactones (PCLs) are used in a wide range of applications due to their properties [1,2]. Both natural and synthetic polymers are used in artificial medical applications such as heart valves, stents, urethral catheters and ureteral stents, cartilage scaffolds, joints, blood vessels, artificial skin manufacturing, artificial kidney/hemodialysis membranes, and in drug delivery nanosystems.

One of its main advantages is biodegradation [3,4], the duration of which may depend on many factors, such as, for example, an aerobic and anaerobic environment [5]. In particular, biodegradable polymeric biomaterials have the great advantage of being degradable and removable once their function is fulfilled. The range of applications is wide, and degradable polymers are used in clinical practice as surgical sutures and implants. The use of polycaprolactone allows for inexpensive implant manufacturing and does not expose the patient to subsequent surgeries stemming from the necessity of its removal [6].

Specific polymers offer a wide variety of possibilities for specific uses in biomedical applications. There are various methods of producing PCL implants, such as solvent casting, foaming, electrospinning, phase separation, or molding [7,8]. The simplest, most profitable and effective method of 3D printing is fused deposition modeling (FDM). This process is based on the extrusion of thermoplastic material through a nozzle using spool filament and on the deposition of layer after layer [9]. 

Polycaprolactone is a biocompatible, nontoxic aliphatic polyester that can be degraded by hydrolysis of its ester linkages in physiological conditions, and it is approved by the FDA for biomedical applications. It is especially very interesting for the preparation of long-term implantable devices and is widely used in long-term implants and controlled drug release applications.

Polycaprolactone possesses some antibacterial properties [10,11]. This particular feature is very important. Tissue damage from surgery or ingestion of foreign bodies further increases susceptibility to infection, activates the host’s defenses, and promotes the production of inflammatory mediators, which are enhanced by bacterial activity and toxins. These types of implants can be used in particular in laryngology, where the surgical field in the area of the nose and sinuses is not perfectly sterile.

Many researchers indicate the need for PCL modifications that increase its bactericidal effectiveness. PCL itself can be a carrier of antibacterial substances [12,13]. Its modifications consist mainly in adding substances with known bactericidal characteristics. These could be copper, silver, zinc oxide, bioglass, or graphene [14,15,16,17,18].

Graphene is an advanced material which successfully finds application in medicine. Biomedical applications of graphene and its composites include its use for gene delivery and small drug molecules. In addition, it is used for biofunctionalization of proteins, anticancer therapy, and as an antimicrobial agent for bone and dental implants. One of its characteristics is antibacterial properties [19]. All aspects of the antibacterial activity of graphene have not yet been fully recognized; however, the antibacterial mechanism of graphene involves both physical and chemical modes of action. Various mechanisms of antimicrobial action of graphene have been proposed, including oxidative stress, (reactive oxygen species produced by graphene inactivate proteins and lipids, preventing bacteria from multiplying), electron transport (it can act as an electron acceptor and abstract electrons from bacterial membrane, which may compromise the membrane integrity), and membrane stress. It has a specific surface that enhances the antibacterial effect by enabling compatible interactions with bacteria membranes. It is believed that the cytotoxicity and inhibition of bacterial proliferation and adhesion are responsible for it [20], just as the direct contact of its sharp edges with the membranes of microbial cells causes the destructive extraction of lipid particles from their interior [21].

Due to its physical and chemical properties and biocompatibility, graphene and graphene oxide can be used as innovative biomedical materials for biodefense, drug distribution in the body, neo-biotherapy, regenerative medicine, and implant surgery. However, graphene-based nanomaterials can be biocompatible or toxic to living cells. The response of biological cells to these nanomaterials depends largely on the number of layers, lateral size, purity, dosage, surface chemistry, and hydrophilicity.

This work presents a comparative analysis of the bactericidal properties of polycaprolactone samples with the addition of varying content of graphene in the form of nanoparticles (graphene nanoplatelets) (GNP). Typical human pathogens—*S. aureus*, *E. coli*, and *C. albicans* were used in the study. The materials intended for research were the filaments sticks manufactured for the 3D printing technique.

## 2. Results

**Figure 1:**
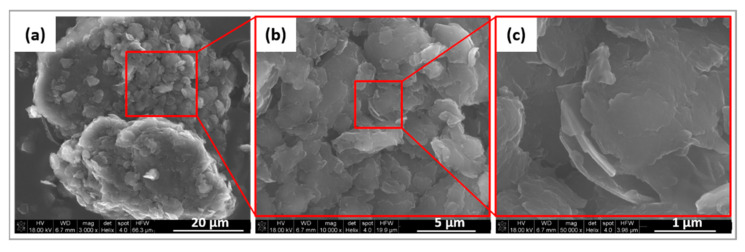
SEM (Scanning Electron Microscope) images of GNP additives.

Graphene nanoplates (GNPs) used as fillers consist of sheets of graphene with a total thickness of several nanometers. SEM images of the introduced GNPs are presented in Figure 1. Plaque-shaped graphene agglomerates are observed. Graphene tends to agglomerate in chunks because of its small particle size, large surface area, and superior particle activity. The morphology is not well-defined, and the agglomerates have an irregular shape with dimensions in the order of a few microns. 

The lowest graphene concentration selected was 0.5 wt%, an intermediate value of 5 wt%, and the maximum concentration used was 10 wt%.

**Figure 2:**
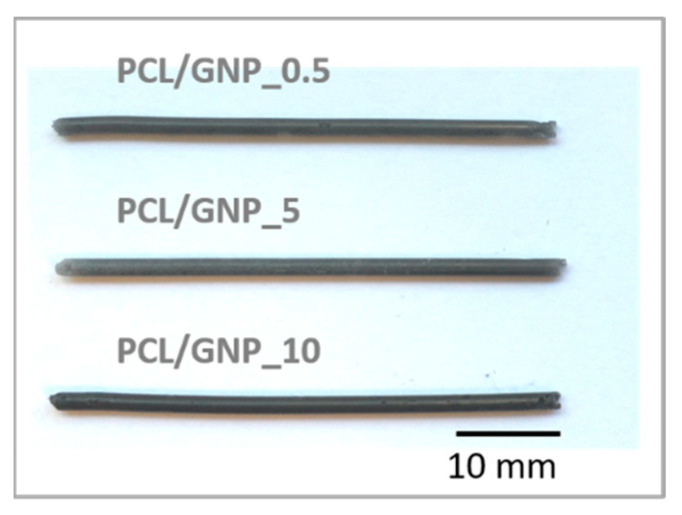
The macroscopic images of obtained composite sticks.

Polycaprolactone blends were prepared containing 0.5, 5, and 10 wt% of graphene content. PCL filaments in the form of composite sticks, for further 3D printing, were made using the injection molding method [22]. Three types of sticks named as PCL/GNP_0.5, PCL/GNP_5, and PCL/GNP_10 were produced, and a pure PCL filament stick was used as a reference material. Macroscopic images of samples are presented in Figure 2. Filaments with a diameter of 1.75 mm and a length of 50 mm were obtained. The samples differ in the intensity of black color.

**Figure 3:**
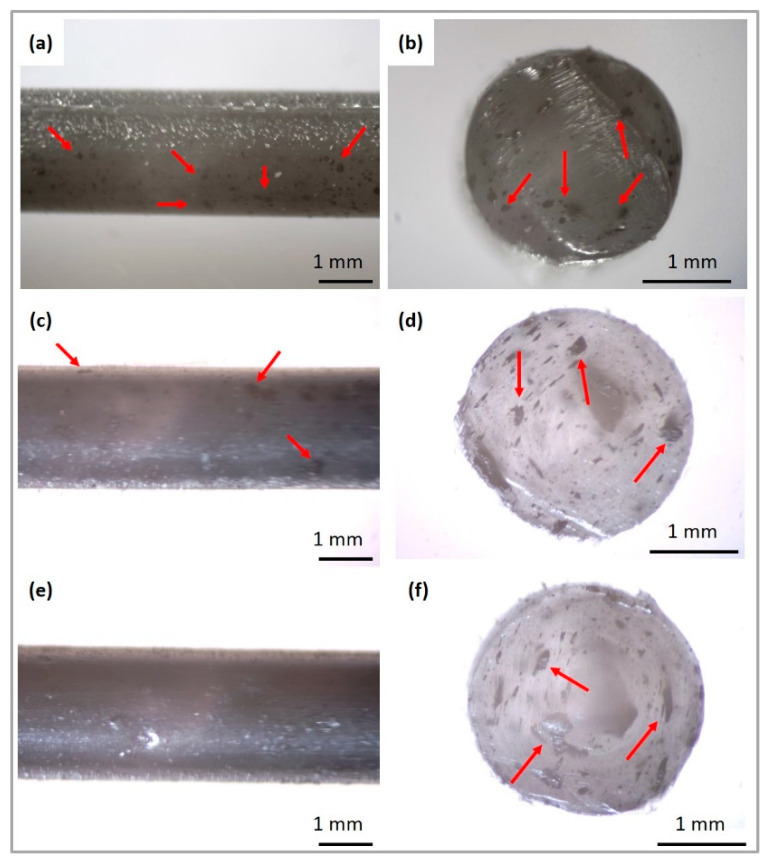
Microscopic images of obtained filaments and its cross section: PCL/GNP_0.5 (**a**,**b**); PCL/GNP_5 (**c**,**d**); PCL/GNP_10 (**e**,**f**). Red arrows indicates graphene concentration.

As opposed to pure PCL, the PCL/GNP sticks exhibited a more irregular surface caused by the GNP present on the surface (Figure 3a,c,e). Investigation of the cross-section of the composite sticks allowed for the analysis of the material in a profile perpendicular to the surface (Figure 3b,d,f). The results indicate that GNP and its agglomerates appeared on the surface and in the cross section of the samples.

**Figure 4:**
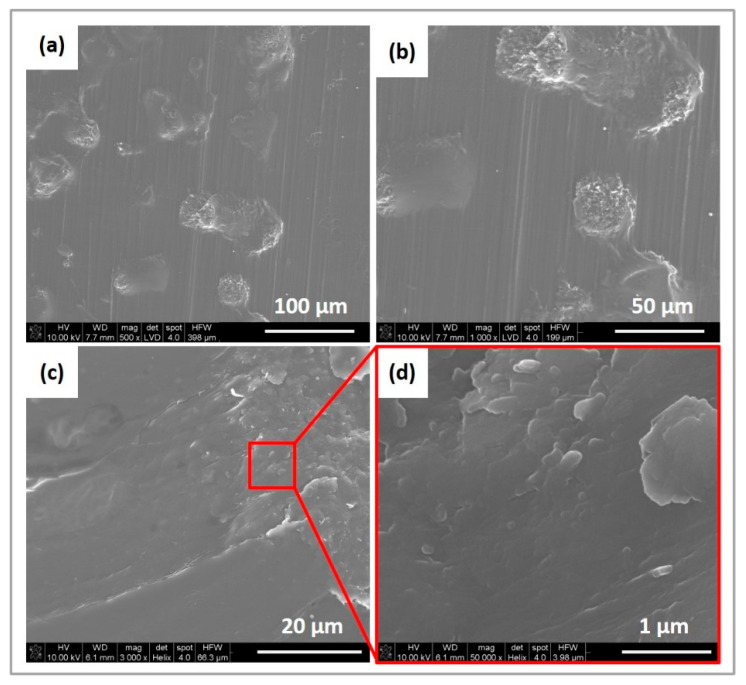
SEM images of PCL_GNP_0.5 stick surface at different magnifications (**a**–**d**).

The SEM (Scanning Electron Microscope) micrographs of the obtained samples were compared with the same magnification X500, X1000, X3000, and X50000. Small agglomerates of graphene formed by sheets were observed on the surface of all modified filaments (Figure 4).

**Figure 5:**
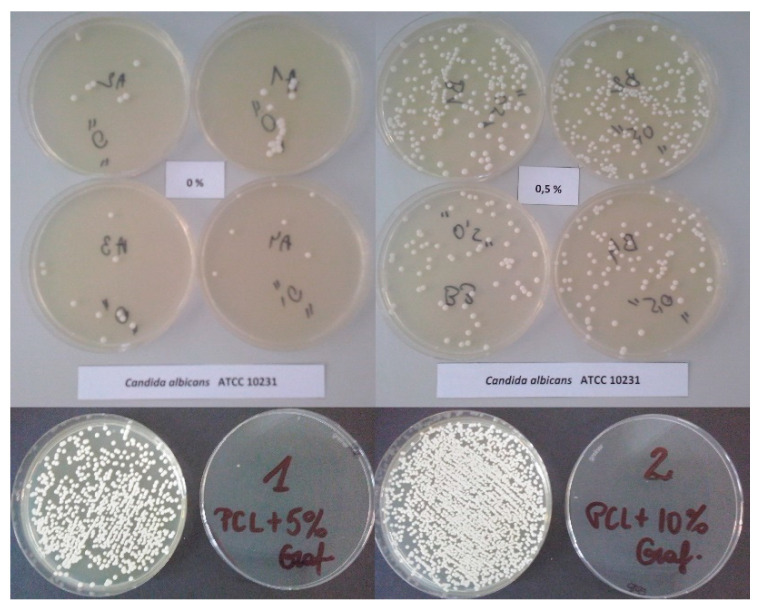
Cultivation of *C. albicans*.

Figure 5 shows the cultivation and growth of *C. albicans*.

Negative controls—normal (no microbial growth). Positive controls—normal (growth of colonies from the reference microbial strains). 

The number of observed microorganisms expressed in the form of the CFU number after 17 h of incubation and the resulting antibacterial efficacy (ABE) are both presented in Table 1.

**Figure 6:**
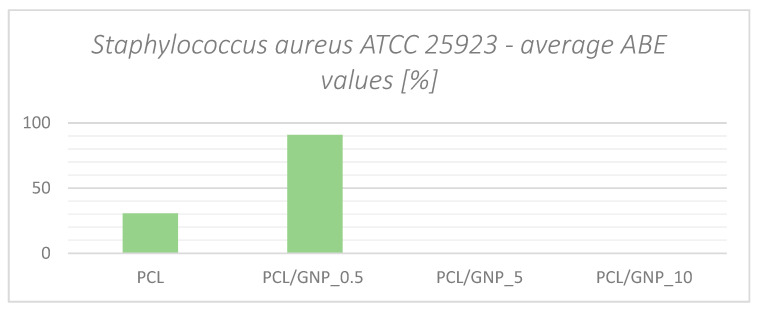
Average ABE values for *S. aureus*.

**Figure 7:**
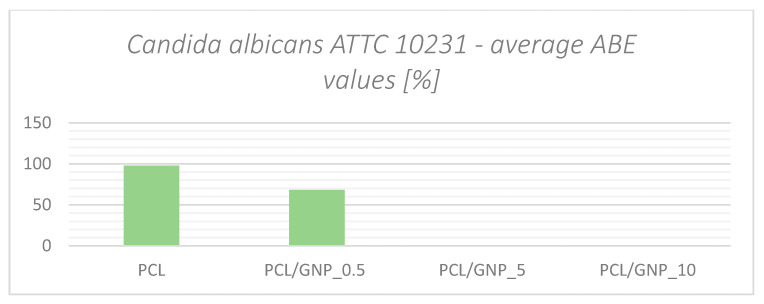
Average ABE values for *C. albicans*.

In the charts (Figure 6 and Figure 7), the summary of the antibacterial efficacy in the form of average ABE values [%] has been presented.

## 3. Discussion

Macro- and microscopic evaluations were performed. The results indicate that graphene nanoparticles were successfully incorporated into the polymer filaments. Higher GNP concentration caused an increase in particle agglomeration. A detailed description of the process parameters and the influence on the properties of the obtained materials was presented in our earlier publication [22]. The results show that at concentrations higher than 10 wt%, the production process becomes difficult. The presence of graphene in the sample was found to improve its mechanical properties: when 0.5 wt% of graphene was added, it was found that the graphene sheets were evenly dispersed in the amorphous phase of the semicrystalline matrix PCL. Initial cell culture studies have shown that scaffolds printed using PCL/GNP filaments support the proliferation of cells. The positive results of preliminary investigation on the composite sticks encouraged us to proceed with the microbiological tests described herein. 

The results show a significant antimicrobial effect against *S. aureus* and *C. albicans*. However, this effect appears to decrease with increasing graphene content. We found no antimicrobial effect at all against *E. coli*.

In the case of *S. aureus*, PCL without additives shows only slight antimicrobial activity at the 30% ABE level. Modification with a small amount of graphene (0.5%) significantly increases the antimicrobial efficacy, reaching a level of 90% ABE. Further increasing the graphene content significantly reduces and suppresses the antimicrobial properties of PCL. Several authors emphasized that graphene exhibits antimicrobial properties in the form of oxides [23]. The structure of graphene may damage the cell membranes of microorganisms and thus eliminate them.

During sample preparation, polymer granules were dry-mixed with GNP powder. It is highly likely that the carbon particles precipitated during the preparation of the culture suspension served as food for the cultured bacteria. However, we concluded that adding only a small amount of graphene (0.5%) to the PCL samples significantly increased their antimicrobial efficacy compared to pure PCL, and further increasing the graphene content effectively reduced the antimicrobial effect.

None of the tested samples showed activity against *E. coli*. In all cases, the inoculated suspensions induced confluent growth of these bacteria. However, a number of researchers confirmed that PCL shows activity against *E. coli*, but only if the polymer is modified, for example, by silver nanoparticles [24,25].

PCL and PCL/GNP_0.5 showed significant antimicrobial activity against *C. albicans*: for PCL, the average ABE was about 98%; when GNP was added to PCL, this value dropped to 68%. Increasing the content of graphene in the samples effectively reduced the antimicrobial activity. This may be due to the fact that carbon particles present in the suspension can become food for bacteria, through a similar process as in the case of *Staphylococcus aureus*.

## 4. Materials and Methods

### Sample Preparation

This research employed polycaprolactone, (PCL, Mn 80 kDa, Sigma Aldrich, St. Louis, MO, USA) and graphene nanoplatelets (polycarboxylate chemically modified graphene nanoplatelets, Sigma Aldrich GNP). In order to produce filaments for further 3D printing, polymer blends with additives and pure PCL granulate were prepared. The polymer granules were dry mixed with GNP powder to obtain 0.5, 5, and 10% by weight of graphene content in the blend. After combining the ingredients, each of the mixtures was mixed in a mechanical mixer for 20 min. Filaments in the form of modified sticks were manufactured in the process of injection molding using a BabyPlast 6/10P (Rambaldi Group, Molteno, Italy) injection molding machine. The parameters of filament production were described in the work [22]. 

**Figure 8:**
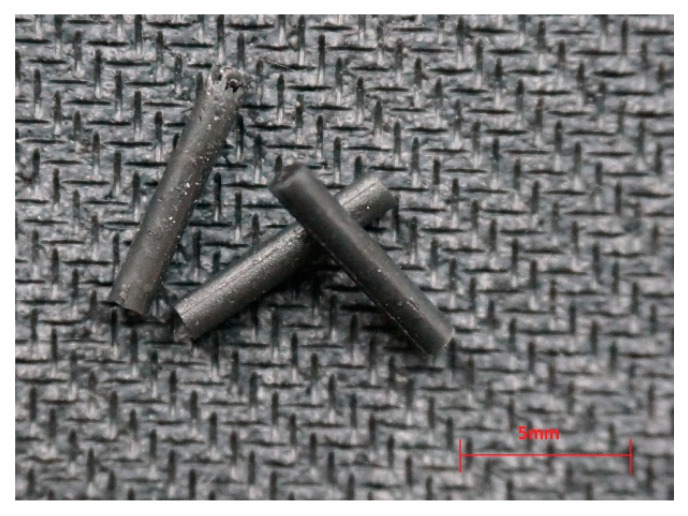
Shape of PCL with 10% GNP samples for microbiological tests.

Microstructural analyses to assess the surface of all samples were carried out in a Nova NanoSEM 200 FEI (Waltham, USA) scanning electron microscope. All the samples were placed on a stainless-steel plate and covered with a carbon thin film by sputtering to avoid the electrostatic charge accumulation. Filaments in the form of sticks were evaluated using a stereomicroscope from the OPTA-TECH company (Warsaw, Poland), equipped with a CMOS 3 camera and OptaViewIS software version 4.3.0.6001. For microbiological tests, filaments were cut and bars with dimensions of 1.75 mm × 6 mm were prepared as shown in Figure 8. All the samples were plasma sterilized.

Polycaprolactone sticks with the addition of 0.5, 5, and 10 wt% graphene were evaluated for bactericidal activity against three selected reference microbial strains:

(1) *Staphylococcus aureus* ATCC 25293
(2) *Escherichia coli* ATCC 25922
(3) *Candida albicans* ATCC 10231

All stages of microbiological tests were carried out in accordance with the principles of asepsis and antiseptics. Each preparation was tested in each replicate with four replicates. A single sample of the test preparation was placed in a 2 mL suspension of the standard strain of the microorganism with a final density of 1.5 × 10^5^ CFU / ml in tryptone water (CFU—colony forming union, i.e., a colony forming unit or single cell of the microorganism). The positive control was the volume 2 mL of a suspension of a given reference strain of microorganism in tryptone water, whereas a negative control was 2 mL of tryptone water. After 17 h of dynamic incubation (Therm-Shaker PST-60HL-4 from bioSan, Riga, Latvia) at 37 °C (*S. aureus* and *E. coli*) or 35 °C (*C. albicans*) from each test sample and control, 20 μL of the suspension was plated on a solid culture medium: Columbia agar with 5% sheep blood (*S. aureus*), Mac Conkey agar (*E. coli*), or Sabouraud agar (*C. albicans*). After 24 h of incubation at 37 °C (*S. aureus* and *E. coli*) or 48 h incubation at 35 °C (*C. albicans*), the observed microbial colonies were counted using the automatic colony counter “aCOLyte” (Synbios, Cambridge, UK) to determine bactericidal efficacy (ABE—antibacterial efficacy) or fungicide (AFE—antifungal efficacy) of the tested preparations [26,27].

## 5. Conclusions

The use of PCL polymers is widespread in modern medicine. Appropriate modifications of this polymer can enhance its basic properties: in this case, the antimicrobial effect. Adding a small amount of graphene can significantly improve these properties.

The results of our research show that increasing the content of graphene in the excipients reduces their bactericidal effect or increases the multiplication of bacteria. It is essential that the newly created biomaterials must always be analyzed in detail. Although the base ingredients separately have good bactericidal properties, their combination may reduce this effect.

The bacteria which were selected in our work, constitute a saprophytic flora in humans [28,29,30]. However, in many cases, these bacteria can cause serious infections, and they often affect people with weakened immunity or those undergoing immunosuppressive therapy. The materials tested can be successfully used in implantology, where a temporary tissue skeleton needs to be created. This is particularly important in cases where the risk of perioperative infection is high, such as in otorhinolaryngologic nasal surgery.

## Figures and Tables

**Table 1:** Overview of the number of microorganisms in individual preparations [CFU] and antibacterial efficacy [ABE] for a given sample.

	*Staphylococcus aureus* ATTC 25923—Initial Density 1.5 × 10^5^ CFU/mL		*Escherichia coli ATTC* 25922—Initial Density 1.5 × 10^5^ CFU/mL		*Candida albicans ATTC* 10231—Initial Density 1.5 × 10^5^ CFU/mL	
Preparation Tested	The Number of CFU/mL after 17 h of Incubation with the Preparation in Individual Samples	Antibacterial Efficacy (ABE) [%] in Individual Samples	The Number of CFU/mL after 17 h of Incubation with the Preparation in Individual Samples	Antibacterial Efficacy (ABE) [%] in Individual Samples	The Number of CFU/mL after 17 h of Incubation with the Preparation in Individual Samples	Antibacterial Efficacy (ABE) [%] in Individual Samples
PCL	63,300	9.57	2.1 × 10^8^—confluent growth obtained for each repeat test	0	1000	95.98
	51,350	26.64		0	500	97.99
	49,750	28.93		0	350	98.59
	2980	57.43		0	300	98.8
PCL/GNP_0.5	12,050	82.78	2.1 × 10^8^—confluent growth obtained for each repeat test	0	11,550	53.61
	8050	88.5		0	10,150	59.24
	4750	93.21		0	5500	77.91
	900	98.71		0	4300	82.73
PCL/GNP_5	7.0 × 10^4^—confluent growth obtained for each repeat test	0	2.1 × 10^8^—confluent growth obtained for each repeat test	0	29,500	0
		0		0	25,500	0
		0		0	25,250	0
		0		0	25,000	0
PCL/GNP_10	7.0 × 10^4^—confluent growth obtained for each repeat test	0	2.1 × 10^8^—confluent growth obtained for each repeat test	0	5.0 × 10^4^—confluent growth obtained for each repeat test	0
		0		0		0
		0		0		0
		0		0		0

## Data Availability

Data is contained within the article.
